# Analysis of risk factors for postoperative complications in hypospadias and construction and validation of a nomogram predictive model

**DOI:** 10.1371/journal.pone.0339188

**Published:** 2025-12-18

**Authors:** Kaiyi Mao, Leibo Wang, Zhouhang Peng, Yuchen Mao, Peng Zhao, Cao Wang, Guangxu Zhou, Zhen Luo, Hongyang Tan

**Affiliations:** 1 Department of Pediatric Surgery, Affiliated Hospital of Zunyi Medical University, Zunyi, China; 2 Department of Pediatric Surgery, Guizhou Children’s Hospital, Zunyi, China; 3 Urology Surgery, Beijing Jishuitan Hospital Guizhou Hospital, Guiyang, China; 4 Department of Emergency, Affiliated Hospital of Zunyi Medical University, Zunyi, China; Instituto Nacional de Ciencias Médicas y Nutrición Salvador Zubiran: Instituto Nacional de Ciencias Medicas y Nutricion Salvador Zubiran, MEXICO

## Abstract

**Background:**

The incidence of postoperative complications in children with hypospadias is notably high; however, research on predictive models for these complications remains limited. This study aims to analyze the factors associated with postoperative complications following hypospadias surgery and to develop a nomogram predictive model for such complications.

**Methods:**

This study included 553 hypospadias patients who underwent surgery at Zunyi Medical University Affiliated Hospital from 1/1/2016–1/1/2023.The patients were randomly divided into training (n = 389) and validation (n = 164) cohorts in a 7:3 ratio. Univariate and multivariate logistic regression analyses were performed on the training cohort to identify risk factors for postoperative complications, which were then used to develop a nomogram prediction model. Finally, the internal validation cohort was used to assess the model’s prognostic accuracy and clinical utility. All statistical analyses were performed using R software(Version 4.2.2).

**Results:**

Multivariate logistic regression analysis of the training cohort identified six independent risk factors for postoperative complications in hypospadias surgery: age (OR=1.02, 95% CI: 1.01–1.03, *P* < 0.001), surgeon’s experience (OR=0.44, 95% CI: 0.26–0.75, *P* = 0.0026), glans width (OR=0.70, 95% CI: 0.59–0.82, *P* < 0.001), length of reconstructed urethra (OR=1.03, 95% CI: 1.01–1.06, *P* = 0.003), hypospadias classification (OR=4.26, 95% CI: 1.96–9.26, *P* < 0.001), and urethral stent retention time (OR=0.25, 95% CI: 0.12–0.52, *P* < 0.001). Based on these factors, a nomogram was constructed. The area under the curve of the nomogram model was 0.800, and after internal validation it was 0.821, indicating good discriminative ability. Furthermore, the model exhibited excellent calibration and high clinical utility.

**Conclusion:**

This study developed and validated a nomogram prediction model for postoperative complications in hypospadias surgery based on six factors: age, surgeon’s experience, glans width, length of reconstructed urethra, hypospadias classification, and urethral stent retention time. It provides a scientific basis for implementing personalized medicine in hypospadias patients in the future.

## Introduction

Hypospadias is a common congenital malformation of the external genitalia, with its incidence gradually increasing in recent years. Over the past 30 years, the incidence of hypospadias has risen by 60%, and approximately 0.2% of male neonates worldwide are affected [1]. In China, the incidence is approximately 9.03 per 10,000 male births [2].Hypospadias typically results from abnormal development of the urethra between the 8th and 17th weeks of gestation [3]. The condition is primarily characterized by an abnormal ventral location of the urethral meatus, penile ventral curvature, and abnormal distribution of the prepuce. In adulthood, hypospadias patients may experience urethral dysfunction, dissatisfaction with penile appearance, sexual dysfunction, and fertility issues, which can severely impact their quality of life [4,5].The only effective treatment for hypospadias is surgical intervention to restore both the appearance and function of the genitalia. Over 300 hypospadias repair techniques exist, including MAGPI, TIP, Onlay, Mathieu, and Duckett. Among these, the most commonly used techniques worldwide are TIP and staged repair [6,7]. However, hypospadias patients face a high risk of postoperative complications, with the incidence ranging from 13% to 56% [8,9]. Common complications include urethral stricture, urethral skin fistula, and recurrent penile curvature. These complications may require secondary or even multiple surgeries, severely impacting both the physical and psychological health of the patients. Additionally, they impose significant financial and emotional burdens on the patients’ families and society. Therefore, early identification of high-risk patients for postoperative complications is critical.

This study retrospectively analyzes the clinical data of hypospadias patients who underwent surgery at Zunyi Medical University Affiliated Hospital. The aim is to identify risk factors for postoperative complications and, based on this, develop and validate a nomogram prediction model for postoperative complications. This will assist pediatric urologists in early identification of high-risk patients and provide evidence-based support for personalized care.

## Materials and methods

### Study population

This is a retrospective study that consecutively collected clinical data from 553 patients who underwent surgical treatment for hypospadias at the Department of Pediatric Surgery, Zunyi Medical University Affiliated Hospital, between 1/1/2016 and 1/1/2023. For the purposes of this research, the date of data access for this study is 28/1/2025.The patients were randomly divided into training (n = 389) and validation (n = 164) cohorts in a 7:3 ratio. Inclusion criteria: 1) First-time urethroplasty at our hospital with a minimum follow-up period of 1 year; 2) Complete clinical data. Exclusion criteria: 1) Incomplete clinical data; 2) History of penile or urethral reconstructive surgery; 3) Abnormal sexual development; 4) Concurrent autoimmune or hematologic disorders; 5) Intellectual developmental disorders; 6) Preoperative testosterone therapy. This study was approved by the Ethics Committee of Zunyi Medical University Affiliated Hospital(approval number: KLL-2024–596). Due to the minimal risk associated with this retrospective study, the Ethics Committee waived the requirement for informed consent.All data has been de-identified.

### Data collection

The candidate predictive variables were categorized into three groups: patient demographic information, penile-related anatomical factors, and surgical-related factors. Patient demographic information included age, body mass index (BMI), and a history of cryptorchidism. Penile-related factors included hypospadias classification, glans width, degree of penile curvature, and urethral plate width. Surgical-related factors included surgeon’s experience, surgical technique, season of surgery, operation time, intraoperative blood loss, length of reconstructed urethra, use of dorsal plication, chordectomy, method of anesthesia, diameter and types of urethral stents, and urethral stent retention time. In total, 19 candidate predictive factors were collected.

### Related definitions

Hypospadias is classified based on the location of the urethral meatus into: distal type, midshaft type, and proximal type [10]. Urethroplasty techniques include: tubularized incised plate urethroplasty (TIP) [11], Onlay island flap urethroplasty [12], Mathieu [13], transverse preputial island flap urethroplasty (Duckett) [14], and Duckett combined with Duplay urethroplasty [15]. Postoperative complications are defined as problems that require surgical correction during follow-up, including urethral or meatal stenosis, urethral skin fistula, glans dehiscence, and recurrent penile ventral curvature. The choice of surgical technique depends on the surgeon’s experience, which is measured by the number of years the surgeon has been performing hypospadias surgeries. All patients underwent urethroplasty with absorbable sutures (PDS Plus Antibacterial (polydioxanone) Suture (6−0)).Anesthesia methods are classified into general anesthesia and combined general anesthesia with dorsal penile nerve block.No caudal or local infiltration anesthesia was used in this cohort. No adrenaline infiltration or tourniquet was applied during any of the procedures.All patients underwent postoperative per-urethral catheterization with silicone-based stents, without the use of suprapubic catheters. Urethral stent types include the Foley double-lumen balloon catheter and the single-lumen non-balloon catheter. Penile curvature is measured using a protractor after achieving a full erection with the Gittes test. Glans width is measured with a ruler at the widest part of the glans before placement of the traction suture and urethral stent during surgery.

### Follow-up

All patients were followed up via outpatient visits and telephone consultations. The follow-up periods were 1, 3, 6, and 12 months after discharge. The presence of postoperative complications within the first year was specifically assessed.

### Statistical analysis

All statistical analyses in this study were performed using R software (Version 4.2.2). First, univariate logistic regression analysis was conducted on the clinical and pathological characteristics of the patients in the training cohort. Variables with statistical significance in the univariate analysis were then included in a multivariate logistic regression analysis to identify independent risk factors for postoperative complications in hypospadias patients and to construct a nomogram prediction model. Internal validation was performed using the validation cohort. The model’s discriminative ability was evaluated by plotting receiver operating characteristic (ROC) curves and calculating the area under the curve (AUC). An AUC value between 0.6 and 0.75 indicates fair predictive performance, 0.75 to 0.85 indicates good predictive performance, and values above 0.85 indicate excellent predictive performance. Calibration curves were also plotted to assess the model’s accuracy, and decision curve analysis (DCA) was used to determine the model’s clinical utility and net benefit. Statistical significance was set at *P* < 0.05.

## Results

### Characteristics of the study population

**Fig 1:**
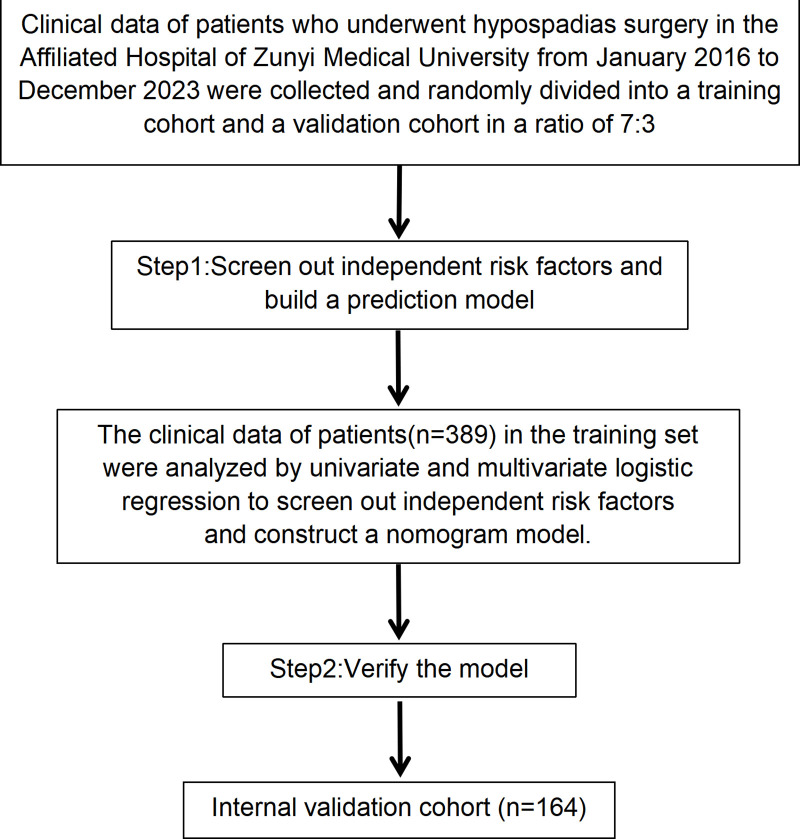
Study flow chart.

A total of 553 patients were included in this study (Fig 1). The average age was 63.19 ± 37.62 months. Among them, 166 patients had distal-type hypospadias, 212 had midshaft-type hypospadias, and 175 had proximal-type hypospadias. All 553 cases were successfully followed up.Average follow-up was 10.13 ± 3.26 months, median follow-up was 12.0 months.Of the 553 patients who underwent hypospadias surgery, 174 developed postoperative complications (31.46%). The complications included urethral skin fistula in 118 cases (21.34%), urethral stenosis in 44 cases (7.96%), and glans dehiscence in 12 cases (2.17%). No cases of recurrent ventral curvature were observed during the follow-up. Patients were randomly divided into training (n = 389) and internal validation (n = 164) cohorts in a 7:3 ratio. In the training cohort, 125 patients (32.13%) experienced postoperative complications, while 49 patients (29.88%) in the validation cohort experienced complications. The clinical characteristics of the two cohorts were compared in detail, as shown in Table 1.

**Table 1:** Characteristics of datasets.

Variables	Training cohort(n = 389)	Internal validation cohort(n = 164)	*P*
Urethral stent retention time(days)			0.602
＜7	49 (12.60)	24 (14.63)	
7-10	273 (70.18)	108 (65.85)	
＞10	67 (17.22)	32 (19.51)	
Season of surgery			0.186
Spring	103 (26.48)	35 (21.34)	
Summer	116 (29.82)	56 (34.15)	
Autumn	53 (13.62)	15 (9.15)	
Winter	117 (30.08)	58 (35.37)	
Diameter of urethral stent			0.316
F6	75 (19.28)	24 (14.63)	
F8	303 (77.89)	137 (83.54)	
F10	11 (2.83)	3 (1.83)	
Operation time(min)			0.488
60≤	70 (17.99)	22 (13.41)	
61-120	241 (61.95)	112 (68.29)	
121-180	66 (16.97)	26 (15.85)	
＞180	12 (3.08)	4 (2.44)	
Intraoperative blood loss(ml)			0.059
＜10	71 (18.25)	17 (10.37)	
10-20	299 (76.86)	140 (85.37)	
＞20	19 (4.88)	7 (4.27)	
Classification of hypospadias			0.481
Distal	121 (31.11)	45 (27.44)	
Middle	143 (36.76)	69 (42.07)	
Proximal	125 (32.13)	50 (30.49)	
Surgical technique			0.433
TIP	173 (44.47)	84 (51.22)	
Duckett	96 (24.68)	33 (20.12)	
Mathieu	87 (22.37)	37 (22.56)	
Onlay	21 (5.40)	8 (4.88)	
Duplay+Duckett	12 (3.08)	2 (1.22)	
Chordectomy			0.689
Yes	226 (58.10)	99 (60.37)	
NO	163 (41.90)	65 (39.63)	
Dorsal Plication			0.397
YES	59 (15.17)	30 (18.52)	
NO	330 (84.83)	132 (81.48)	
History of cryptorchidism			0.500
NO	366 (94.09)	155 (94.51)	
Unilateral cryptorchidism	13 (3.34)	3 (1.83)	
Bilateral cryptorchidism	10 (2.57)	6 (3.66)	
Degree of penile curvature			0.325
＜15°	211 (54.24)	88 (53.66)	
15-30°	71 (18.25)	38 (23.17)	
＞30°	107 (27.51)	38 (23.17)	
Surgeon’s experience(years)			0.165
≤4	164 (42.16)	55 (33.54)	
5-8	178 (45.76)	87 (53.05)	
≥9	47 (12.08)	22 (13.41)	
Types of urethral stents			0.943
Double chamber	304 (78.15)	127 (77.44)	
Single chamber	85 (21.85)	37 (22.56)	
Method of anesthesia			0.815
General anesthetics	339 (87.15)	141 (85.98)	
General anesthetics + Penile nerve block	50 (12.85)	23 (14.02)	
Urethral plate width(mm)	6.116 (1.809)	6.098 (1.728)	0.913
Age (months)	61.082 (36.335)	68.189 (40.166)	0.042
BMI(Kg/m^2^)	15.330 (3.149)	15.341 (3.121)	0.978
Length of reconstructed urethra (mm)	25.499 (13.863)	25.220 (12.928)	0.826
Glans width(mm)	14.440 (1.751)	15.067 (1.436)	＜0.001

BMI, body mass index.

### Selection of risk factors

Univariate logistic regression analysis was used to evaluate the relationship between candidate predictive factors and postoperative complications in hypospadias patients. The statistically significant variables identified in univariate analysis were included in multivariate logistic regression analysis. Six independent risk factors were found to be associated with the risk of postoperative complications in hypospadias patients. These factors were: age(OR=1.02, 95% CI: 1.01–1.03, *P* < 0.001), surgeon’s experience (OR=0.44, 95% CI: 0.26–0.75, *P* = 0.0026), glans width (OR=0.7, 95% CI: 0.59–0.82, *P* < 0.001), length of reconstructed urethra (OR=1.03, 95% CI: 1.01–1.06, *P* = 0.003), hypospadias type (OR=4.26, 95% CI: 1.96–9.26, *P* < 0.001), and duration of urethral stent retention (OR=0.25, 95% CI: 0.12–0.52, *P* < 0.001). These results are summarized in Table 2.

**Table 2:** Univariate and multivariate logistic regression analysis.

Variables	Univariate analysis		*P*	Multivariate analysis		*P*
	OR	95%CI		OR	95%CI	
Method of anesthesia						
General anesthetics						
General anesthetics + Penile nerve block	1.64	0.89-3	0.11			
Intraoperative blood loss(ml)						
＜10						
10-20	1.21	0.68-2.14	0.52			
＞20	2.29	0.81-6.48	0.12			
Chordectomy						
Yes						
NO	0.66	0.43-1.03	0.07			
Degree of penile curvature						
＜15°						
15-30°	1.64	0.93-2.88	0.09			
＞30°	1.33	0.81-2.19	0.26			
Diameter of urethral stent						
F6						
F8	1.25	0.71-2.18	0.44			
F10	2.14	0.59-7.78	0.25			
Dorsal Plication						
YES						
NO	0.91	0.51-1.64	0.75			
Surgeon’s experience(years)						
≤4						
5-8	0.56	0.36-0.89	0.01	0.44	0.26-0.75	0.0026
≥9	0.52	0.25-1.08	0.08	0.4	0.17-0.94	0.0365
History of cryptorchidism						
NO						
Unilateral cryptorchidism	0.95	0.29-3.13	0.93			
Bilateral cryptorchidism	1.42	0.39-5.12	0.59			
Classification of hypospadias						
Distal						
Middle	1.85	1.02-3.35	0.04	1.63	0.84-3.18	0.1506
Proximal	5	2.78-8.98	<0.001	4.26	1.96-9.26	＜0.001
Operation time(min)						
60≤						
61-120	1.46	0.8-2.69	0.22			
121-180	1.9	0.91-3.98	0.09			
＞180	3.12	0.89-10.95	0.08			
Season of surgery						
Spring						
Summer	0.99	0.56-1.74	0.97			
Autumn	0.96	0.47-1.95	0.91			
Winter	0.9	0.51-1.59	0.72			
Urethral stent retention time(days)						
＜7						
7-10	0.28	0.15-0.53	<0.001	0.25	0.12-0.52	＜0.001
＞10	0.37	0.17-0.79	0.01	0.26	0.11-0.64	0.0034
Surgical technique						
TIP						
Duckett	1.44	0.86-2.43	0.17			
Mathieu	0.7	0.39-1.26	0.24			
Onlay	0.88	0.32-2.40	0.8			
Duplay+Duckett	2.2	0.68-7.15	0.19			
Types of urethral stents						
Double chamber						
Single chamber	0.68	0.4-1.17	0.16			
Urethral plate width(mm)	0.95	0.84-1.07	0.39			
Length of reconstructed urethra (mm)	1.05	1.03-1.06	<0.001	1.03	1.01-1.06	0.003
BMI(Kg/m^2^)	0.93	0.86-1	0.06			
Age(months)	1.01	1-1.01	0.03	1.02	1.01-1.03	<0.001
Glans width(mm)	0.81	0.72-0.92	<0.001	0.7	0.59-0.82	<0.001

OR,Odds ratio;CI,Confidence interval;BMI,Body mass index.

### Construction of the nomogram prediction model

**Fig 2:**
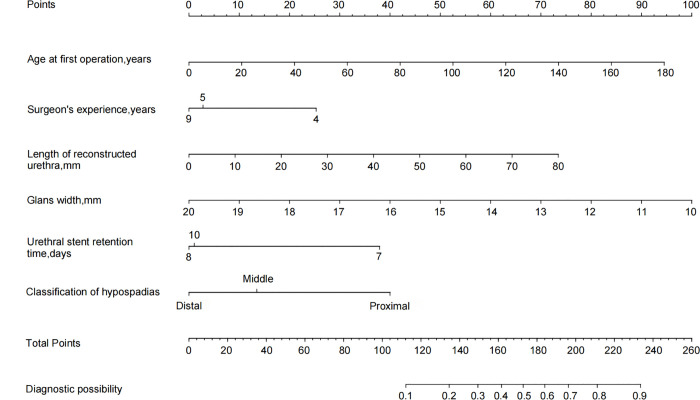
Nomogram for predicting the risk of complications after hypospadias surgery.

The six independent risk factors identified in the multivariate logistic regression analysis were used as independent variables. A multivariate logistic regression model was established to describe the functional relationship between these predictors and postoperative complications. The regression coefficients for each predictor were quantified and assigned values. The contribution of each predictor to the risk of postoperative complications was calculated and presented as a nomogram, as shown in Fig 2.

### Internal validation

**Fig 3:**
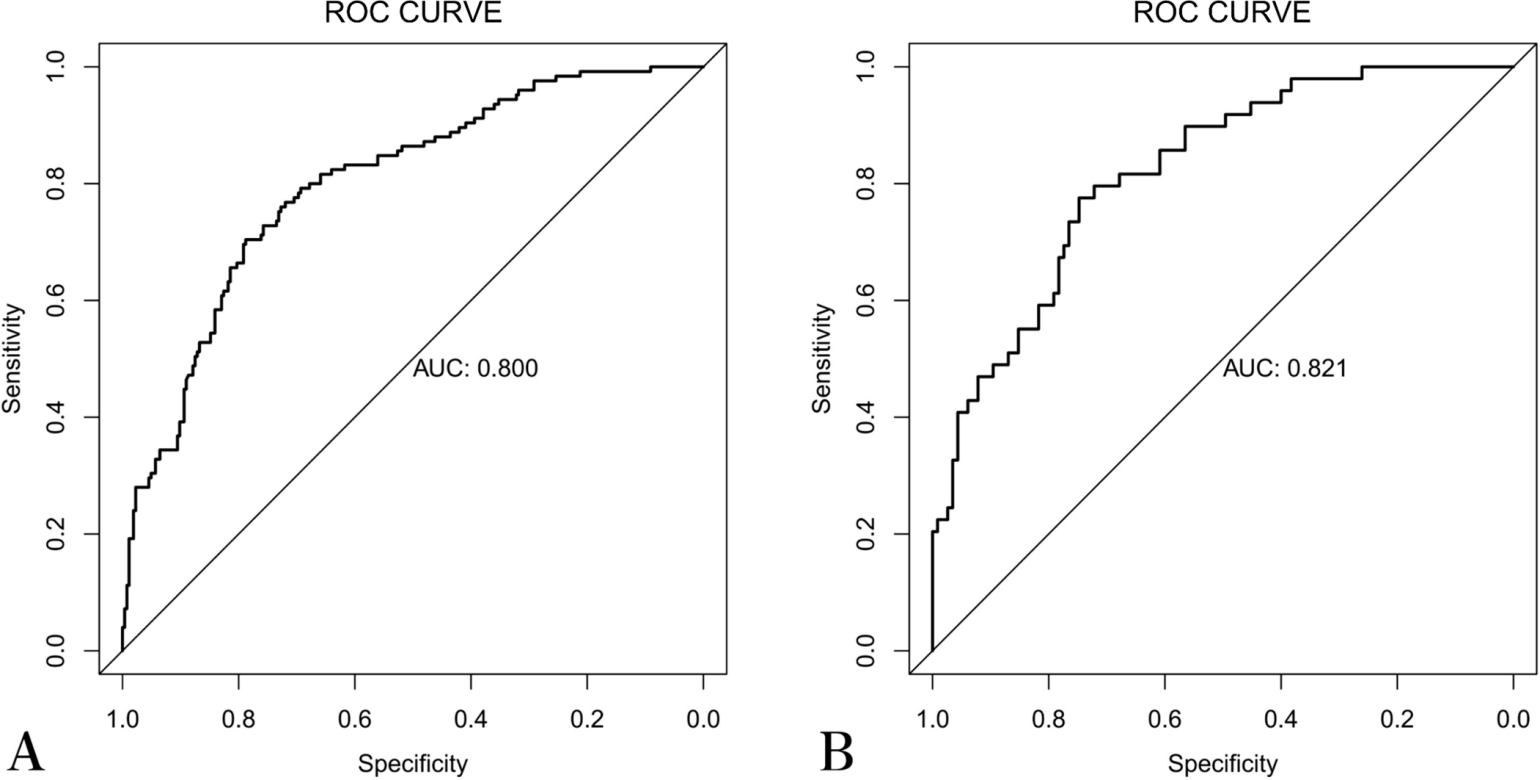
Receiver operating characteristic analysis of nomogram model based on training cohort (A), internal validation cohort (B).

**Fig 4:**
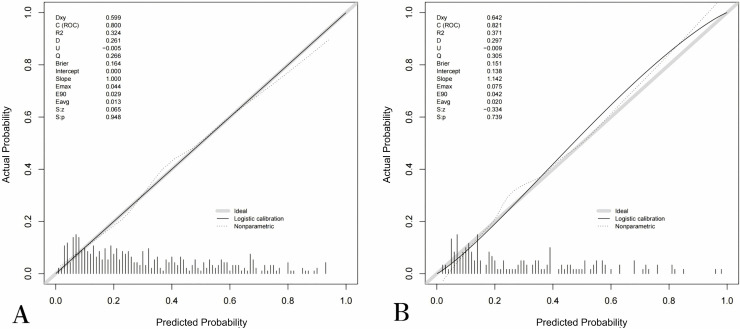
The calibration curves of the nomogram model based on training cohort (A), internal validation cohort (B).

**Fig 5:**
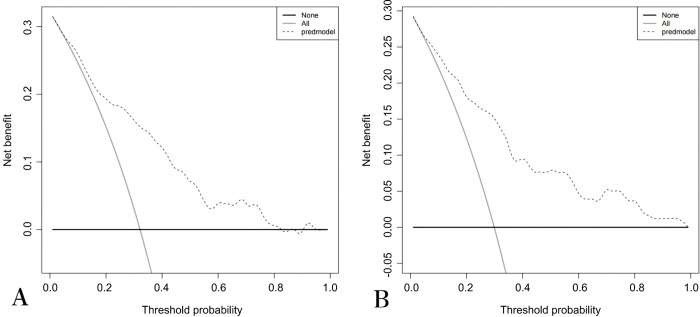
Decision curve analysis of nomogram model based on training cohort (A), internal validation cohort (B).

In both the training and internal validation cohorts, the model demonstrated good discriminative ability. The AUC for the model was 0.800 in the training cohort (Fig 3A) and 0.821 in the internal validation cohort (Fig 3B), indicating strong discriminatory power. The calibration curve showed good concordance between the predicted risk of postoperative complications and the actual occurrence of complications, as shown in Fig 4A and 4B. The DCA (Figs 5A and 5B) was used to assess the clinical utility of the proposed nomogram model. According to the DCA, the model generated higher net benefits across most threshold probability ranges.

## Discussion

Hypospadias is a common genital malformation in male children, with surgery being the only effective treatment option. Despite continuous advancements in surgical techniques for hypospadias, the incidence of postoperative complications remains high, and many of these complications require corrective surgery. This not only has a significant psychological impact on the affected children and their families but also severely affects their quality of life. Therefore, identifying high-risk factors associated with postoperative complications is crucial, as it helps clinicians provide targeted interventions. In this study, we developed and validated a risk prediction model for postoperative complications in hypospadias surgery based on retrospective single-center data. Through univariate and multivariate logistic regression analyses of the training cohort, we identified that age, hypospadias type, length of reconstructed urethra, glans width, surgeon’s experience, and urethral stent retention time were independent risk factors for postoperative complications. These six independent risk factors were then used to construct a nomogram prediction model for postoperative complications, which was validated in the validation cohort. The performance of the nomogram model in the validation cohort was satisfactory, with an AUC of 0.821. Combined with the results from the calibration curve and DCA, the model demonstrated excellent discriminatory ability, calibration, and clinical utility, indicating its potential for widespread application and use.

This study found that age is an independent risk factor for postoperative complications in hypospadias. As the child’s age increases, the risk of postoperative complications also rises (OR = 1.02), which is consistent with previous studies [16,17]. Da Silva et al. observed a significant increase in the concentration of total collagen with aging, along with a progressive reduction in the elastic fiber content of the urethral plate. [18]. Additionally, studies have shown that the expression levels of pro-inflammatory factors (such as IL-6 and IL-8) produced by fibroblasts in the foreskin tissue also increase with age [19]. These changes may impair the healing capacity of the surgical site, thereby increasing the risk of complications. The American Academy of Pediatrics recommends an ideal surgical age of 6–12 months, and the European Association of Urology suggests performing surgery between 6–18 months. In China, the surgical age typically ranges from 6 to 30 months [20]. However, in this study, the median surgical age of the patients was 52 months, which is significantly higher than the internationally recommended surgical age. This discrepancy may be attributed to the fact that most of the patients in our center come from rural areas, where there is a lack of awareness of the condition and insufficient emphasis on regular pediatric check-ups, leading to delayed visits for treatment. Therefore, increasing public education and awareness about hypospadias is of utmost importance.

Previous studies [21–23] have shown that the type of hypospadias and the length of the reconstructed urethra are closely related to postoperative complications. In our study, both of these factors were confirmed as independent risk factors for postoperative complications in hypospadias. Clinically, these two factors are positively correlated, meaning that the closer the urethral opening is to the perineum, the longer the urethral defect, and consequently, the longer the urethra needs to be reconstructed. The longer the reconstructed urethra, the higher the risk of complications. For patients with proximal hypospadias, the incidence of reoperation following TIP surgery is high [24]. In Duckett surgery, for every 1 cm increase in the length of the reconstructed urethral plate, the risk of postoperative complications increases 3.506-fold [25]. Proximal hypospadias is typically associated with more complex anatomical abnormalities, and the longer urethral reconstruction required means that more flaps or tissue coverage are needed to form the new urethra. This not only requires a richer blood supply but also results in greater tension on the newly reconstructed urethra during the repair process, making wound healing more difficult and increasing the risk of complications. This scenario demands a higher level of surgical skill from the surgeon.

This study found that the surgeon’s experience (≥5 years) is a protective factor for postoperative complications in hypospadias surgery. The proficiency of surgical technique directly affects the quality of the surgery and the incidence of complications. Urethroplasty requires a high level of skill, especially during the urethral reconstruction and anastomosis process, where meticulous operation is critical to reducing postoperative complications. Previous studies [26,27] have reported that as the number of surgeries performed by a surgeon increases, the likelihood of complications significantly decreases. Additionally, some studies have shown that pediatric urologists who have received specialized training exhibit a clear learning curve when performing hypospadias repairs, with the incidence of complications notably decreasing as their experience grows [28]. These findings align with the conclusions of this study. Experienced surgeons are better able to assess and select the appropriate surgical approach when managing patients with proximal hypospadias or severe anatomical abnormalities. Higher surgical proficiency allows them to avoid excessive tension, injury to surrounding tissues, and other surgical risks. Therefore, for complex hypospadias cases, a graded management approach should be adopted, with experienced surgeons taking the lead in surgery to ensure high surgical quality. For beginners, guidance from senior surgeons can help shorten the learning curve.

Although surgical technique is often discussed in relation to outcomes, it was not an independent predictor in our multivariate analysis. This suggests that technique choice may reflect anatomical complexity and surgeon familiarity more than outcome itself, highlighting the need for individualized surgical planning.

Postoperative placement of a urethral stent has been consistently shown to be a key factor in reducing the incidence of complications following hypospadias surgery [27]. However, there is no consensus on the optimal duration for stent placement. Zhou G et al. suggested that the duration of urethral stent placement does not significantly affect the occurrence of postoperative complications in proximal hypospadias [22]. Nonetheless, clinical guidelines generally recommend that the stent remain in place for at least 7 days to minimize the risk of complications [29]. Our study also supports this perspective, demonstrating that a stent retention period of ≥7 days serves as a protective factor against postoperative complications in hypospadias surgery. This is likely due to the fact that the edema or inflammation at the site of the anastomosis between the newly formed urethra and the original urethra may not have fully resolved after surgery. Early removal of the catheter could increase resistance to urine flow at this site or cause extravasation around the urethral anastomosis, potentially leading to complications.

The relationship between glans width and the occurrence of complications after hypospadias surgery remains inconclusive. Faasse MA et al. suggested that glans width is not a significant factor influencing postoperative complications [30]. In their study, the complication rate was 16% for children with a glans width <14 mm, while it was 15% for those with a larger glans. However, this finding contradicts other studies, some of which identify glans width as an independent risk factor for complications after hypospadias surgery [23,31–33]. Notably, Bush et al. reported that a glans width <14 mm is an independent risk factor for postoperative complications (OR 0.77; 95% CI 0.65–0.90) and is negatively correlated with urethroplasty complications [33]. For every 1 mm increase in glans size, the risk of complications decreases by 23%. Our study also observed this trend (OR 0.70; 95% CI 0.59–0.82). This phenomenon may be attributed to the increased tension when suturing the smaller glans during glansplasty, which could impair wound healing and lead to glans dehiscence or urinary fistulas. Moreover, a smaller glans increases the likelihood of urethral stricture due to the relatively smaller urethra. In contrast, a larger glans provides more tissue for urethroplasty, facilitating more precise surgical manipulation and reducing tension during glans and urethra suturing. To mitigate the effects of a small glans, preoperative androgen stimulation or delaying surgery until puberty may be considered to increase glans width. However, the routine use of preoperative androgen stimulation remains controversial, as androgens are believed to inhibit skin repair, delay healing, and increase inflammation in both acute and chronic settings.

Research on predictive models for complications after hypospadias surgery is limited. Previous models primarily included mild to moderate hypospadias patients and focused on a narrow range of predictive variables, excluding intraoperative factors, perioperative urethral stenting, and penile anatomical parameters. The AUC for this models was 0.76 [34]. Additionally, another model aimed at predicting urethral stricture after hypospadias surgery did not incorporate penile anatomical parameters, and the selection of candidate variables was also limited, focusing solely on a single postoperative complication, with an AUC of 0.79 [35]. Both models are likely to overlook significant clinical predictors.

To our knowledge, this is the first study to develop a new predictive model that integrates patient general conditions, perioperative factors, intraoperative variables, and penile anatomical parameters. The model includes 19 candidate variables, expanding the scope of prediction. The AUC in the training cohort and internal validation cohort were 0.80 and 0.82, respectively, demonstrating better discrimination and offering more practical clinical guidance. This model has clinical value not only for preoperative and postoperative management but also in helping clinicians communicate complex data to families in a more understandable risk assessment format. This can aid families in better understanding the potential challenges their child may face after surgery, reduce anxiety caused by information asymmetry, and enhance family involvement and trust.However, this study has several limitations. First, the data were derived from retrospective medical records, which may introduce information bias. Second, as this is a single-center retrospective study, there may be selection bias in the sample. For instance, our center primarily advocates for single-stage surgery, and only in a few exceptional cases, typically involving complex anatomical issues, do we consider performing a two-stage surgery. Therefore, this study only analyzes the outcomes of single-stage surgery. This exclusion may limit the generalizability of our findings to cases requiring two-stage repair and affect the external applicability of this model in other regions or healthcare institutions. Third, the study lacks long-term follow-up data, such as the collection of cases of recurrent penile curvature,which may be due to insufficient follow-up duration.Fourth,the lack of information regarding postoperative adjunctive medications, such as laxatives and antimuscarinics drugs, may pose a potential confounding factor.

Future research should focus on multi-center data validation, integration of long-term follow-up data and postoperative adjunctive medications, inclusion of cases with staged surgeries, and dynamic updates of the model.

### Conclusions

In conclusion, this study developed and validated a predictive model for postoperative complications in hypospadias based on six factors: age, surgeon’s experience, glans width, length of the reconstructed urethra, classification of hypospadias, and urethral stent retention time. The model exhibited strong discriminatory ability, with an AUC of 0.800 in the training cohort and 0.821 in the validation cohort. The calibration curve demonstrated excellent concordance between predicted and observed outcomes, underscoring the model’s reliability. This model demonstrates significant potential in guiding personalized treatment strategies and improving outcomes for patients with hypospadias, thus facilitating more precise clinical decision-making in the future.

## Supporting information

S1 Data All data used for the analysis in this article. (XLSX)
